# Development of antibacterial collagen membranes with optimal silver nanoparticle content for periodontal regeneration

**DOI:** 10.1038/s41598-024-57951-w

**Published:** 2024-03-27

**Authors:** Sara Takallu, Farshad Kakian, Abdollah Bazargani, Hooman Khorshidi, Esmaeil Mirzaei

**Affiliations:** 1 Department of Medical Nanotechnology, School of Advanced Medical Sciences and Technologies, Shiraz University of Medical Sciences, Shiraz, Iran; 2 Department of Bacteriology & Virology, School of Medicine, Shiraz University of Medical Sciences, Shiraz, Iran; 3 Department of Periodontology, School of Dentistry, Shiraz University of Medical Sciences, Shiraz, Iran

**Keywords:** Periodontitis, Silver nanoparticle, Antibacterial activity, Periodontal bacteria, Collagen membrane, Guided tissue regeneration, Materials science, Nanoscience and technology

## Abstract

The effective control of pathogenic bacteria is crucial in the restoration of periodontal tissue affected by periodontitis. Guided tissue regeneration (GTR) membranes are commonly used to aid in the repair of periodontal defects. Therefore, there is a clear advantage in developing antibacterial periodontal membranes that can effectively eliminate infections and promote tissue regeneration. This study aimed to create a collagen membrane with optimal content of silver nanoparticles (AgNPs) for effective antibacterial properties and minimal toxicity to mammalian cells. Ascorbic acid-reduced AgNPs were incorporated into collagen at the ratio of 0.5%, 1%, 2%, and 3% (based on total dry weight). Collagen/AgNPs hydrogels were compressed and freeze-dried to form membranes and then were characterized. Antibacterial activity was tested against *Fusobacterium nucleatum* and *Enterococcus faecalis*, and membrane cytocompatibility was accomplished on human gingival fibroblasts. Membranes with 2% and 3% AgNPs exhibited significant antibacterial activity, while 1% showed minimal activity and 0.5% and 0% showed none. HGF cells on the 3% AgNPs membrane had poor viability, proliferation, and adhesion, but 0%, 0.5%, 1%, and 2% AgNPs membranes showed desirable cellular behavior. In conclusion, the collagen membrane with 2% AgNPs demonstrated both antibacterial capacity and excellent cytocompatibility, making it a promising choice for periodontal treatments, especially in GTR approaches.

## Introduction

Periodontitis is a chronic and prevalent inflammatory disease caused by bacterial infection and the subsequent formation of biofilm. This gum disease is characterized by the progressive destruction of the tissues surrounding the teeth. In cases of severe periodontitis, there is significant damage to the alveolar bone and cementum, ultimately leading to tooth loss^1,2^. Inhibiting inflammation and effectively controlling infection by removing microbial biofilms are the primary goals of periodontitis treatment^3^. Infection control can be achieved using several ways, such as mechanical debridement of plaque and biofilms and systemic and topical antimicrobial agent administration^4^. Nevertheless, in severe periodontitis, removing the infection alone is insufficient, and periodontal tissue repair does not occur spontaneously^5^.

Periodontal regenerative and reconstructive therapies have received widespread attention in the last few decades. The most successful therapeutic approach to date has been guided periodontal regeneration, which includes guided tissue regeneration/guided bone regeneration (GTR/GBR). The use of bioactive and bioresorbable scaffolds promotes cell attachment, proliferation, and specific differentiation, sustaining the formation of new healthy tissues with appropriate texture and biomechanical competence^6,7^. Collagen, a natural polymer, due to its attributes such as biocompatibility, biodegradability, and low antigenicity, is often utilized in medical applications^8^ and as a scaffold is a preferred biomaterial in the fields of regenerative medicine and tissue engineering^9,10^. In addition, to collagen's role in cellular functions such as adhesion, proliferation, and cell differentiation, collagen-based membranes have received special attention in GTR/GBR approaches^11,12^.

Bacterial infections and dental plaque formation are often accompanied by periodontal disease and cause regeneration interference. Previous research has shown that GTR membrane failure occurs most frequently in patients with high levels of periodontal pathogens. Consequently, there is a need to develop periodontal membranes designed for controlling and eliminating infections^13–16^. Therefore, effective control of dental plaque is one of the main to successful periodontal treatment using GTR membranes. Antibiotics are essential for stopping bacterial growth, but antibiotic resistance is a problem for public health. This has encouraged biomedical research into new, highly effective preventative measures and treatment options, with antimicrobial properties^17–19^. Several ingredients with antibacterial and antimicrobial properties can serve as alternatives to conventional antibiotics. Certain natural substances have antibacterial properties, including garlic, ginger, honey, echinacea, goldenseal, clove, and oregano^20^. Also, antimicrobial peptides (AMPs) are a class of small peptides that widely exist in nature and are an important part of the innate immune system. They have a wide range of inhibitory effects against bacteria, fungi, parasites, and viruses. AMPs are divided into subgroups based on their amino acid composition and structure, and most of them have the ability to kill microbial pathogens directly, while others act indirectly by modulating the host defense systems^21^.

Nanotechnology has also significantly contributed to the treatment of periodontitis through various antimicrobial and restorative approaches^22^. Materials with a size between 1 and 100 nm, known as nanoparticles, have different physicochemical properties from their bulk counterparts due to their high surface-to-volume ratio^23^. Specialized nanoparticles, with nanometer particle sizes, can be engineered to target oral tissues, including periodontium. These nanoparticles offer the potential for targeted drug delivery, leading to better inhibition of bacterial growth, inflammatory modulation, and early tissue resolution in periodontitis^24^. For instance, nanoparticles can be engineered to target oral tissues, including the periodontium, for targeted drug delivery. They can be loaded with various antibiotics, such as metronidazole, chlorhexidine, and nystatin, to distribute to periodontal tissues and treat fungal infections and oral mucositis^25^. Nanotechnology enables the development of nano-scale biosensors that can be used to diagnose periodontal disorders. These sensors can identify substances found in bodily fluids such as saliva, blood, and gingival crevicular fluid^26^. Dentists can control nanorobots using onboard computers, transmitting signals for specific treatment techniques, and providing a precise and controlled approach to periodontal treatment^27^. Nanohydroxyapatite (nHA) has shown promise in the treatment of periodontitis. Research has demonstrated that applying subgingival nano-hydroxyapatite powder with an air abrasion device combined with scaling and root planning increases clinical attachment gain, indicating its potential efficacy in periodontal treatment^28^. Moreover, when combined with collagen membranes, nHA has demonstrated a significant improvement in periodontal indices, reducing dentinal hypersensitivity and reconstructing periodontal bone deficiencies. Similar to this, nHA's superior osteoinductive potential and capacity to enhance bone-to-implant integration have made it a popular choice for applications in restorative, regenerative, and preventative dentistry, including implantology and periodontal regeneration^29^. Furthermore, the osteoconductive and osteoinductive properties of titanium (Ti) substrates can be enhanced by calcium ion-exchanged nanosized EMT zeolites (Ca^2+^-EMT). After these nanoscale EMT zeolites were synthesized and characterized, the researchers spin-coated them onto Ti surfaces. According to the study, the Ca^2+^-EMT enclosed in the sodalite cage was found to improve the hydrophilicity and roughness of the EMT zeolite^30^.

Recent advancements in the field of nanotechnology have enabled the production of nanoparticles, particularly metal nanoparticles, as antibacterial agents^31^. A variety of materials, including titanium dioxide, zinc oxide, gold, and silver, have been reported to produce nanoparticles with antibacterial activity against a broad spectrum of pathogenic microorganisms^32^. Small dimensions of nanoparticles enable targeted drug delivery to specific tissues, cells, or pathogens within the periodontal pockets^33^. They also demonstrate antibacterial capabilities by rupturing bacterial cell membranes, which eliminates microbes. They are useful in the treatment of periodontitis because of their specific and powerful antibacterial action, which may also assist in minimizing the development of antibiotic resistance^25^. Furthermore, nanotechnologies have been developed to improve antimicrobial performance in antimicrobial photodynamic therapy (aPDT). These technologies target the delivery of hydrophobic photosensitizers into microorganisms, aiming to reach deep pockets of oral infections. Different nanostructured materials are designed to generate reactive oxygen species (ROS) using UV, visible, and NIR light. This approach has shown potential in dentistry, particularly for deep pocket infections, demonstrating its potential for effective treatment^34^.

Among these nanomaterials, AgNPs exhibit outstanding antimicrobial activity against a wide range of bacteria, including both Gram-positive and Gram-negative bacteria as well as bacteria that are resistant to antibiotics^35,36^. Although the mechanism of action of AgNPs in killing bacteria is still unknown, two main hypotheses have been proposed: direct interaction of nanoparticles with bacterial membranes and release of silver ions^37,38^. According to studies focusing on the oral cavity, AgNPs have an antibacterial effect by hindering the growth of several oral bacteria, such as *S. mutans*, *S. oralis*^39^, and *P. gingivalis*^40^. As well as researchers synthesized nanosized EMT zeolites with silver ion exchanging to inhibit biofilm formation in dental adhesives. The zeolites were used to form single-species biofilms against cariogenic pathogens *Streptococcus mutans*, *Streptococcus gordonii*, and *Streptococcus sanguinis*. The adhesives with Ag^+^-EMT zeolites showed remarkable antibacterial properties, making them potential for anti-biofilm and anti-caries clinical applications^41^. Therefore, incorporating silver nanoparticles in a GTR membrane can be very successful in regenerating periodontal tissue. However, high levels of Ag^+^, have the potential to cause some cell toxicity^42,43^. Therefore, one of the factors that need to be carefully controlled to achieve high antibacterial activity and good cytocompatibility is the concentration of AgNPs.

Collagen is commonly used in tissue regeneration due to its excellent biocompatibility and wide distribution in tissues. It is the main structural protein in the human body, offering low immunogenicity, a porous structure, good permeability, biocompatibility, and biodegradability^44^. Collagen-based scaffolds are widely used in periodontal regeneration due to their various benefits and applications. These scaffolds contribute to the success of guided tissue regeneration and guided bone regeneration. Collagen membranes act as an inhibitory barrier against fibroblast or soft tissue ingrowth, allowing a suitable niche for bone regeneration, particularly in alveolar bone and ridge defects^45^. Collagen induces proper proliferation and differentiation of cells involved in regeneration, contributing to the success of GTR/GBR^46^. Polymeric materials, such as collagen, are employed for the construction of the GTR membranes to regulate degradation rates and promote bioactivity^47^. Studies have demonstrated that collagen scaffolds laden with human periodontal ligament fibroblasts promote periodontal tissue regeneration in animal models. In vivo studies have shown that collagen-based scaffolds can achieve complete periodontium regeneration, including periodontal ligaments and cementum, via stem cell recruitment and activation^48^.

To the best of our knowledge, no previous studies have reported on collagen membranes with the optimal percentage of AgNPs for guided tissue regeneration in periodontitis. Therefore, the objective of this study was to develop a collagen membrane with varying percentages of AgNPs and determine the ideal AgNPs content that exhibits effective antibacterial activity while remaining non-toxic to cells for GTR applications. AgNPs were synthesized using an environmentally friendly method and subsequently incorporated into a collagen hydrogel. The resulting nanocomposite hydrogels were then compressed and freeze-dried to produce nanocomposite membranes. The physicochemical properties of these membranes were assessed. In addition, the in vitro antibacterial activity of the membranes, containing different percentages of AgNPs, was analyzed against oral disease-related pathogenic bacteria, including *Fusobacterium nucleatum* and *Enterococcus faecalis*. Finally, the cytocompatibility of the membranes was evaluated through in vitro testing on human gingival fibroblast cells.

## Materials and methods

### Materials

Silver nitrate (AgNO_3_), ascorbic acid (C_6_H_8_O_6_), trisodium citrate (Na_3_C_6_H_5_O_7_), sodium hydroxide (NaOH), fluorescein diacetate (FDA), propidium iodide (PI) and acetic acid were purchased from Sigma-Aldrich. Type 1 collagen was extracted from rat tails. Human gingival fibroblast (HGF) cells were purchased from the Pasteur Institute of Iran. Dulbecco’s modified Eagle’s medium (DMEM), fetal bovine serum (FBS), penicillin–streptomycin, and 2-4,5 dimethyl-thiazole 2,5-diphenyl-tetrazolium bromide (MTT) were purchased from Gibco. *Fusobacterium nucleatum*, isolated from a clinical sample,^49^, was obtained from the Department of Bacteriology and Virology, School of Medicine, Shiraz University of Medical Sciences, Shiraz, IR Iran, and *Enterococcus faecalis* (*E. faecalis*, ATCC 29212) was purchased from the Pasteur Institute of Iran.

### Preparation of AgNPs

Ag nanoparticles were synthesized using a previously described procedure^50^—the—synthesis involved utilizing ascorbic acid as a reducing agent and sodium citrate as a stabilizing agent. Specifically, a 64 ml aqueous solution containing 6.72 mg of ascorbic acid and 49.52 mg of sodium citrate was adjusted to a pH value of 10.5 by adding a 5 M NaOH solution. Subsequently, 0.64 ml of a 0.1 M aqueous solution of AgNO_3_ was introduced to the solution and stirred in a 30 °C water bath for 30 min. The addition of the AgNO_3_ resulted in a noticeable change in the solution's color, transitioning from colorless to brown.

### Preparation of AgNPs incorporated collagen membranes

**Figure 1:**
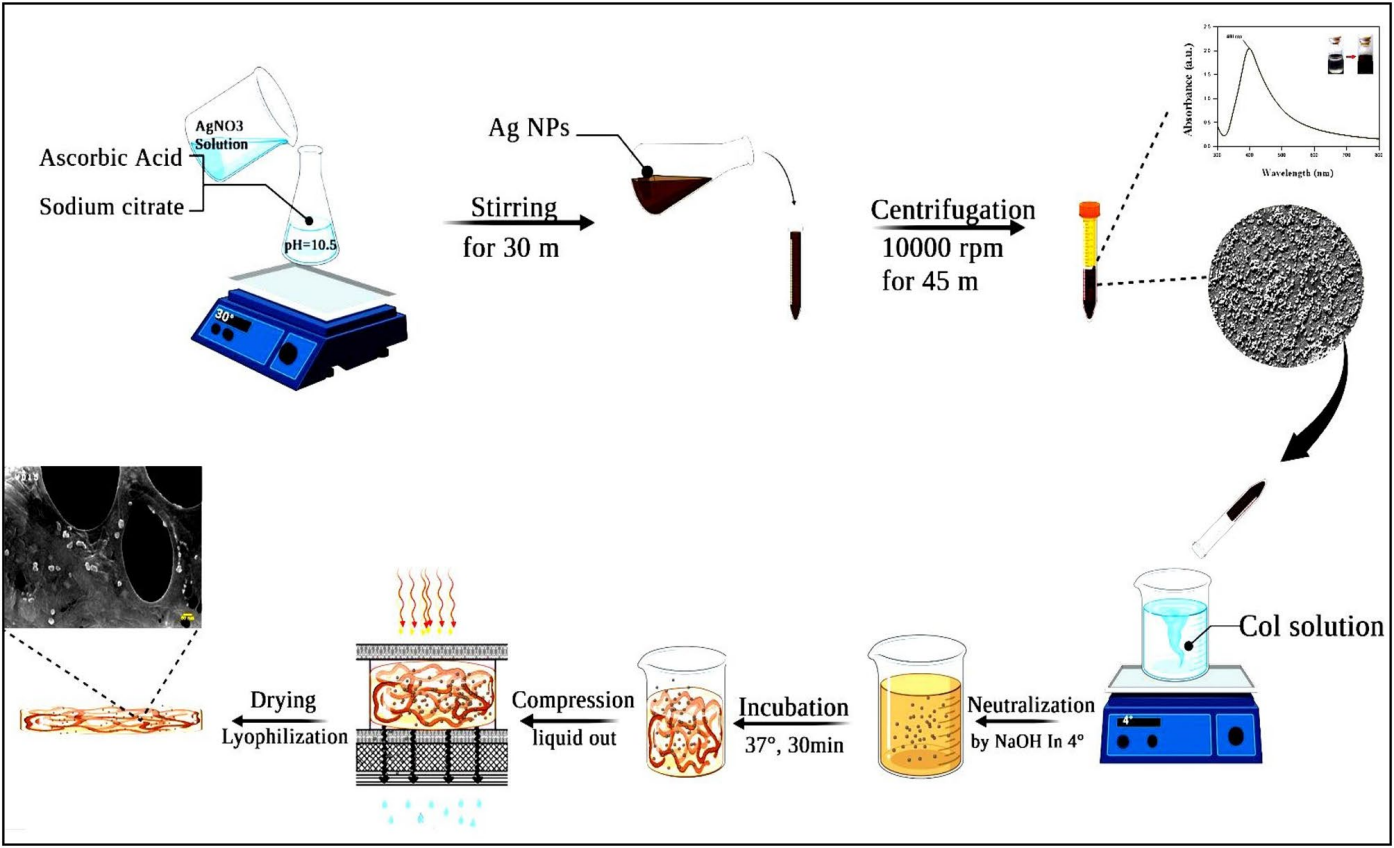
Schematic illustration of AgNPs synthesis and collagen/Ag membrane preparation.

To prepare the collagen/Ag membranes, a 3 mg/mL collagen solution in 0.5 M acetic acid was initially prepared and placed on a stirrer. Once the collagen had dissolved, the solution was supplemented with 0.5%, 1%, 2%, and 3% AgNPs (based on total dry weight). The pH of the solution was then neutralized by adding a 5 M NaOH solution, and the mixture was incubated at 37 °C for 30 min to allow for hydrogel formation. Following this, the hydrogel underwent plastic compression, and the resulting compressed membrane was dried using a freeze-dryer. The collagen membranes containing 0%, 0.5%, 1%, 2%, and 3% AgNPs were designated as Col/Ag-0, Col/Ag-0.5, Col/Ag-1, Col/Ag-2, and Col/Ag-3, respectively. A schematic illustration depicting the synthesis of AgNPs and the collagen/Ag membranes can be found in Fig. 1.

### Characterizations

The synthesis of AgNPs was analyzed using a UV–Vis spectrophotometer, which operated in the wavelength range of 300–800 nm. The morphology of the AgNPs and collagen/Ag membrane was observed through FE-SEM with a Zeiss Sigma 300 microscope. For investigating the crystallinity of the samples, X-ray diffraction (XRD) was performed on the AgNPs and collagen/Ag membranes using an ASENWARE AW-XDM300 X-ray diffractometer equipped with Cu Kα radiation sources (λ = 0.154 nm) operating at 18 kV. The diffraction angle was set to range from 2θ = 10° to 80°.

To determine the chemical structure of the collagen membrane and collagen/Ag membranes, Fourier Transform Infrared (FTIR) analysis was conducted in the spectral range of 400–4000 cm^−1^ using Bruker Vertex 70 and Bruker Tensor II instruments.

The tensile strength of the collagen and optimized collagen/Ag membranes was measured via a stress–strain test. Membranes with 1 mm in thickness were cut into 35 mm in length and 10 mm in width and stretched at a speed of 5 mm/min under a 200 N sensor using a mechanical testing machine (SANTAM/STM-150, Iran). The as-prepared specimens were placed and fixed between the inner sides of grips, covered by the specimen holders, and drawn at the crosshead rate of 5 mm/min up to the mechanical failure. The test was conducted 5 times for each sample and the data were reported as mean ± SD.

### Antibacterial activity of collagen/Ag membranes

The antibacterial activity of collagen/Ag membranes was assessed through several tests, including the disk-diffusion test, membrane-bacteria co-culture test, and bacterial infiltration tests, as outlined below:

### Disk-diffusion test

The disk-diffusion test, also known as the inhibition zone method, was used as a semi-quantitative test to evaluate the antibacterial activity of membranes. Before this test, the samples were cut to a diameter of 6 mm and sterilized with ultraviolet rays for 30 min. The standard antibiotic disks of ampicillin and gentamycin were used as positive controls. Suspension of *Fusobacterium nucleatum* and *Enterococcus faecalis* bacteria with turbidity equivalent to 0.5 McFarland (1.5 × 10^8^ CFU/ml) was prepared. Then the bacterial colony was removed with a swap and dissolved in physiological serum. After dewatering, suspensions were uniformly spread on the Müller Hinton agar. Then the rounded samples were gently placed on the surface of the agar and incubated at 37 °C for 24 h. After 24 h incubation at 37 °C, the bacterial-free zones surrounding the growth were observed with a digital camera and accurately measured with a millimeter ruler, the test was repeated three times^51^.

### Membrane-bacteria co-culture test

For this test, the collagen/Ag membranes were cut into 1 cm diameter disks and placed at the bottom of a 24-well plate (n = 3). Subsequently, 1 ml of McFarland's bacterial suspension (10^7^ CFU/mL) was added to the membranes, and the plate was incubated at 37 °C for 24 h. Next, the contents of the wells were transferred to sterile tubes containing 5 ml of PBS and vortexed for 10 min. Serial dilutions were then prepared by transferring 100 µl from each tube to the first well (well 12) of a 96-well plate containing Muller Hinton broth medium. The serial dilution was continued from well 12 to the subsequent wells. After preparing the dilution series, cultures were performed on Mueller Hinton agar for each well. Specifically, 100 µl from each well was taken and spread onto Mueller Hinton agar plates, which were then incubated for an additional 24 h. Following incubation, the colonies on each plate were counted to determine the bacterial count^52,53^.

### Bacterial infiltration test

Bacterial infiltration through the Col/Ag-0, Col/Ag-0.5, Col/Ag-1, Col/Ag-2, and Col/Ag-3 membranes was investigated by evaluating the morphology of bacteria on the membranes. After co-culturing the samples with the bacteria, they were collected and washed three times with PBS (pH 7.4). Subsequently, the membranes were fixed with 2.5% (v/v) glutaraldehyde at 4 °C for 2 h. Following fixation, the samples were washed with phosphate-buffered saline (PBS, pH = 7.4) and dehydrated using a graded series of ethanol concentrations (30%, 50%, 70%, and 90%) for 15 min each, followed by 100% ethanol for 1 h. Once the membranes were dried, the bacterial morphologies on the membrane surface were observed using scanning electron microscopy^51^.

### In vitro cellular behavior (cytocompatibility)

The cellular behavior of HGF cells, including viability, proliferation, and cell morphology, was assessed using live/dead assay, MTT assay, and SEM imaging, respectively.

### The MTT assay

The cytocompatibility of the membranes in terms of cell proliferation was assessed using HGF cells through the MTT assay. HGF cells were seeded onto sterilized circular membranes in 96-well plates at a concentration of 5 × 10^3^ cells/well. The plates were then incubated for 1, 3, and 7 days in triplicate. At the designated time points, the stock solution was removed, and MTT solution with a concentration of 0.5 g/L in PBS was added to each well. The cells were further incubated for 4 h. Subsequently, the optical density (OD) of the formazan product was measured at 570 nm using a microplate reader. The OD values were recorded for the cell/membrane groups, and a control group consisting of cells without membranes was also included for comparison. This assay allowed for the evaluation of cell proliferation on the membranes and provided insights into their cytocompatibility.

### The live/dead staining

To assess the viability of cells cultured on the membranes, a live/dead staining assay was conducted using fluorescein diacetate (FDA) and propidium iodide (PI). HGF cells were cultured on sterilized circular membranes in a 48-well plate at a concentration of 2 × 10^4^ cells/ml for 1, 3, and 7 days (n = 4). At each time point, the media were removed, and the samples were gently washed with PBS. Subsequently, the cells were stained with fluorescein diacetate (5 mg/ml) to visualize live cells (green fluorescence) and propidium iodide (3 mg/ml) to detect dead cells (red fluorescence). The staining was performed for 5 min, away from light. Following staining, the samples were rinsed with PBS and observed using inverted fluorescence microscopy (Varian Cary Eclipse, Agilent Technologies, USA). Images were captured to visualize and analyze the distribution of live and dead cells on the membranes. This staining assay provided valuable information regarding the viability of cells cultured on the membranes over different periods.

### Cell attachment

**Figure 2:**
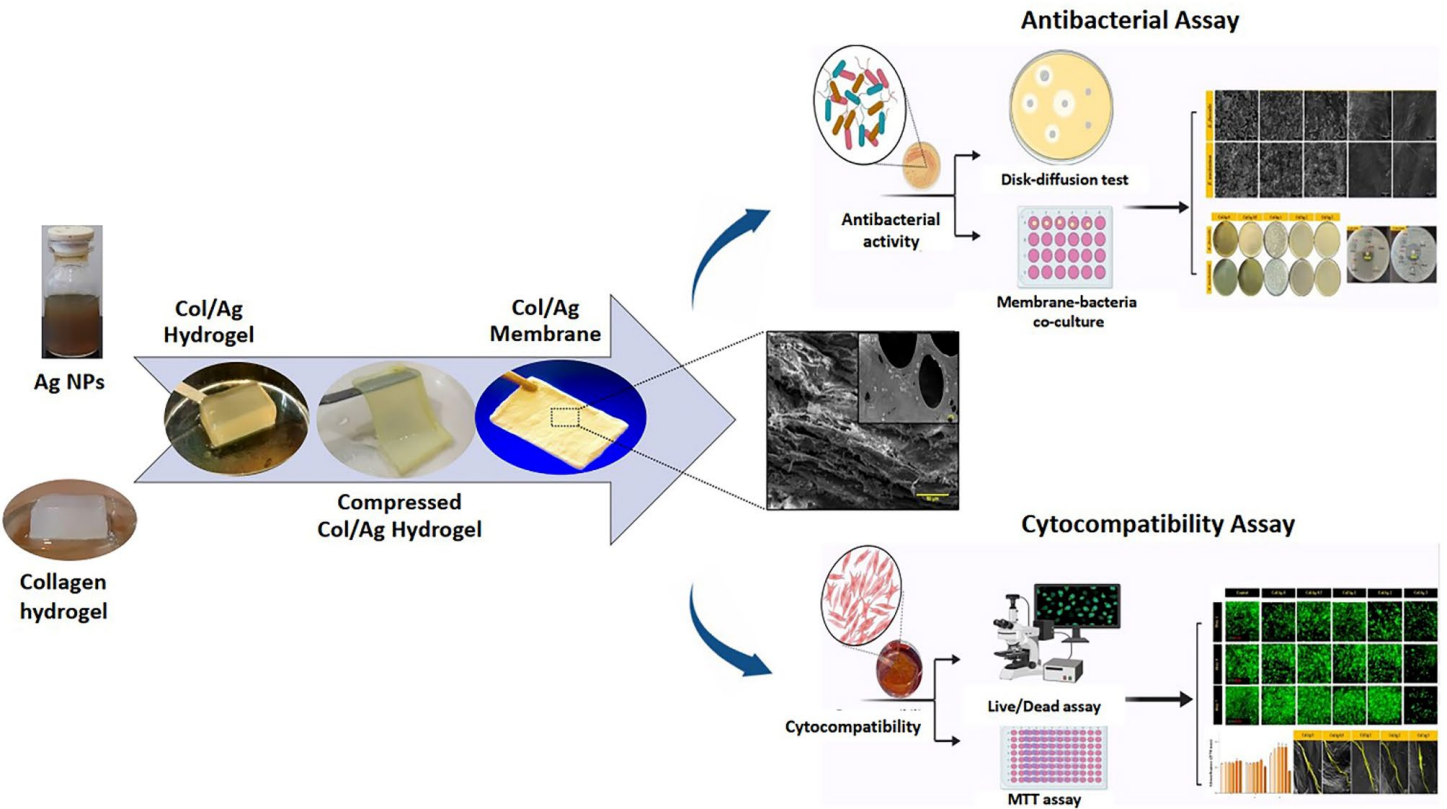
Schematic illustration of collage/Ag membrane and analysis of antimicrobial activity and membrane biocompatibility.

The cell attachment was observed by taking SEM images of the membrane 5 days after cell culture. For this, the adhered cells with the membrane were treated with 2.5% glutaraldehyde, followed by thorough washing in PBS two times. Then the samples were dehydrated in an ethanol-graded series (50, 60, 70, 80, 90, and 100%) for 10 min each and allowed to dry on a clean petri dish at room temperature. The surface of the cell-adhered membrane was observed by SEM after gold coating. Schematic illustration of collage/Ag membrane and overview of the antimicrobial and cell compatibility tests is shown in Fig. 2.

### Statistical analysis

All experiments were carried out in at least three separate studies, and data were presented as mean ± standard deviation (SD). GraphPad Prism Software was used for the analysis. To determine statistical significance, student's t-test and one-way ANOVA along with Tukey's post hoc test were conducted. Any differences that had a p-value of less than 0.05 were considered significant.

## Results

### UV–Vis spectrophotometry and morphological characteristics of AgNPs

**Figure 3:**
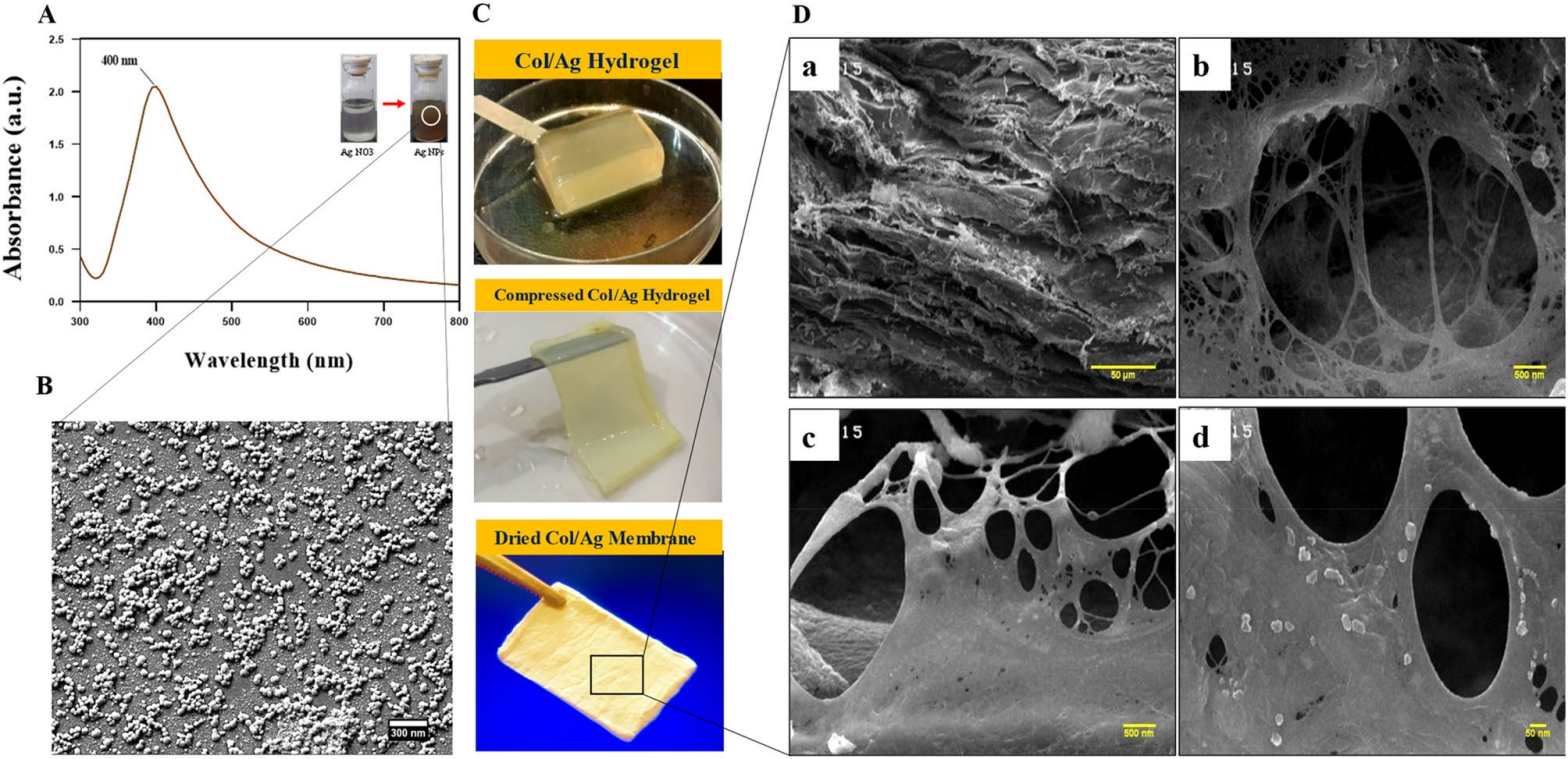
(**A**) UV–Vis spectra of silver nanoparticles (AgNPs), (**B**) SEM image of AgNPs, (**C**) digital images of AgNPs incorporated collagen (Col/Ag) hydrogel, Col/Ag compressed hydrogel, and Col/Ag membrane and (**D**) SEM micrographs of Col/Ag membrane at different magnifications**.**

AgNPs were synthesized using ascorbic acid as a reducing agent and citric acid as a stabilizer, making the process environmentally friendly. The formation of AgNPs was visually confirmed by a color change from colorless to brown. In Fig. 3A, the UV–Vis absorption spectra of the AgNPs aqueous solution after 30 min of reaction time are depicted. The spectra exhibited a surface plasmon resonance (SPR) band at 400 nm, which is characteristic of silver colloids. It is worth noting that spherical AgNPs typically exhibit the highest absorption between 380 and 450 nm, as indicated by previous studies^54–56^. Therefore, the presence of the plasmon band within this range confirms the spherical shape of the nanoparticles. This finding was further supported by FE-SEM imaging, which was employed to determine the size and morphology of the AgNPs. The images in Fig. 3B revealed that the AgNPs are in the nanoscale, with an average diameter of 30 nm and a roughly spherical shape.

### Morphological characterization of membranes

Figure 3C showcases digital images of the Col/Ag hydrogel, Col/Ag compressed hydrogel, and Col/Ag dry membrane. The cross-sectional SEM micrographs of the collagen/Ag membrane are presented in Fig. 3D. Upon examining the SEM images of the Col/Ag membrane at four different magnifications, it becomes evident that AgNPs are uniformly embedded throughout the collagen membrane. Moreover, the inner cross-sectional regions of the Col/Ag membrane exhibit a heterogeneous interconnected structure with open porosity, featuring micro-scale pore sizes, as depicted in the SEM micrographs. Notably, the majority of mammalian cells fall within this size range, and the porous network on these membranes facilitates angiogenesis, cell growth, and cell proliferation^57^. The incorporation of nanoparticles into the acidic collagen solution, followed by neutralization and collagen hydrogel formation, ensures the even dispersion of silver nanoparticles within the collagen hydrogel. The aminophilic nature of AgNPs enables their physical binding to collagen molecules^58^. In summary, the Col/Ag membrane is a collagen-based hydrogel that promotes tissue regeneration due to its porous network, which supports angiogenesis, cell growth, and cell proliferation^59^.

### X-Ray diffraction

**Figure 4:**
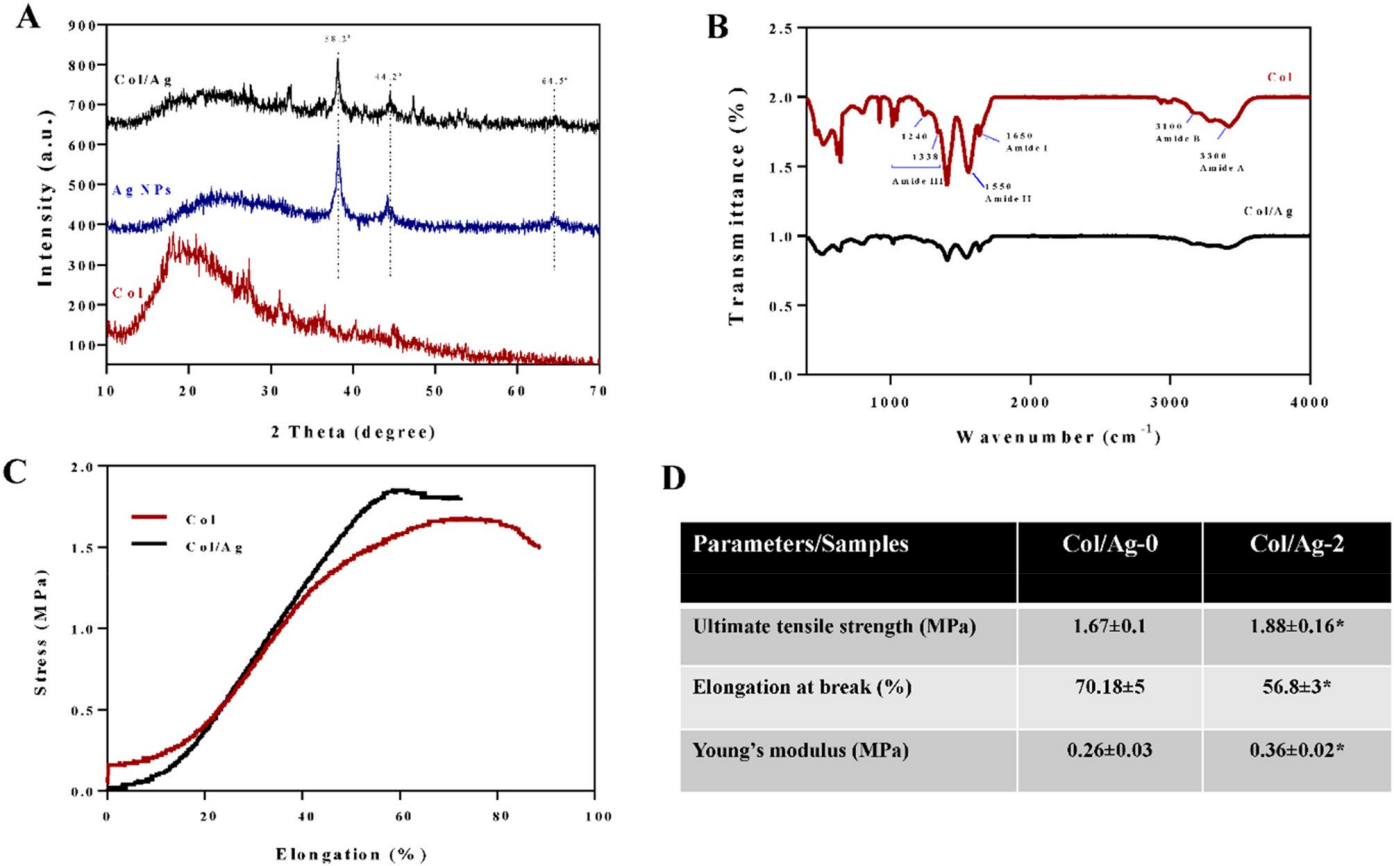
(**A**) X-ray diffraction patterns of collagen membrane (Col), silver nanoparticles (AgNPs), and AgNPs incorporated collagen membrane (Col/Ag). (**B**) FT-IR spectral analysis of collagen and collagen/Ag membrane. Representative stress–strain curves (**C**) and the corresponding mechanical parameters (**D**) of Col and Col/Ag membranes. *Indicates statistically significant difference in comparison with Col membrane (p < 0.05).

Figure 4A displays the XRD patterns of AgNPs, Col membrane, and Col/Ag membrane. X-ray diffraction analysis provided conclusive evidence of the successful synthesis of AgNPs. The presence of peaks at 2θ = 38.2°, 44.2°, and 64.5° in the XRD spectrum of AgNPs confirms the crystalline nature of the synthesized nanoparticles.

### Fourier-transform infrared spectroscopy

The FTIR spectra of collagen and collagen/Ag membrane are shown in Fig. 4B. The collagen spectrum showed five distinct amide bands; the band at 3300 cm^−1^ declared amide A of collagen attributed with N–H stretching, while the band at 3100 cm^−1^ is associated with N–H stretching vibration and C–H stretching. The peaks of amide I (1650 cm^−1^) are caused by C=O stretching vibrations, amide II (1550 cm^−1^) is caused by N–H bending vibrations, and amide III (1400–1200 cm^−1^) seems to be proof that collagen has a triple helical structure^60,61^. Changes in the intensity and position of amide bands are accompanied by changes in the secondary structure of collagen^62^. The presence of AgNPs did not change the position of the bands, but it caused a decrease in the intensity of the amide bands. The decrease in the intensity of the amide bands suggests that the incorporation of AgNPs into the collagen membrane may have caused some disruption to the secondary structure of collagen. This disruption could be due to interactions between the AgNPs and the collagen molecules, which could affect the hydrogen bonding and other interactions that contribute to the stability of the collagen structure^63^.

### Mechanical properties

An ideal GTR membrane for guided periodontal tissue repair should have favorable mechanical properties. The tensile stresses of Col and Col/Ag-2 were average at 1.67 ± 0.1 MPa and 1.88 ± 0.16 MPa, the breaking strains at 70.18 ± 5% and 56.8 ± 3%, and the Young’s modulus was at 0.26 ± 0.03 MP and 0.36 ± 0.02 MPa, respectively (Fig. 4C and D). The incorporation of 2% Ag NPs into the Col membrane significantly affects Young’s modulus of the Col membrane. Furthermore, the ultimate tensile strength increased significantly, and elongation at break decreased significantly. The tensile strength of the membranes in this study is comparable to the tensile strength of OSSIX® Plus and Bio-Gide, two commercialized collagen barrier membranes^64^.

### Antibacterial activity evaluation

The antimicrobial efficacy of compressed collagen membranes containing varying amounts of AgNPs was examined against both a Gram-positive bacterial strain (*Enterococcus faecalis*) and a Gram-negative bacterial strain (*Fusobacterium nucleatum*). The antimicrobial effectiveness was evaluated using the inhibition zone method, membrane-bacteria co-culture, and SEM imaging of bacterial morphology on the membranes.

### Inhibition zone method

**Figure 5:**
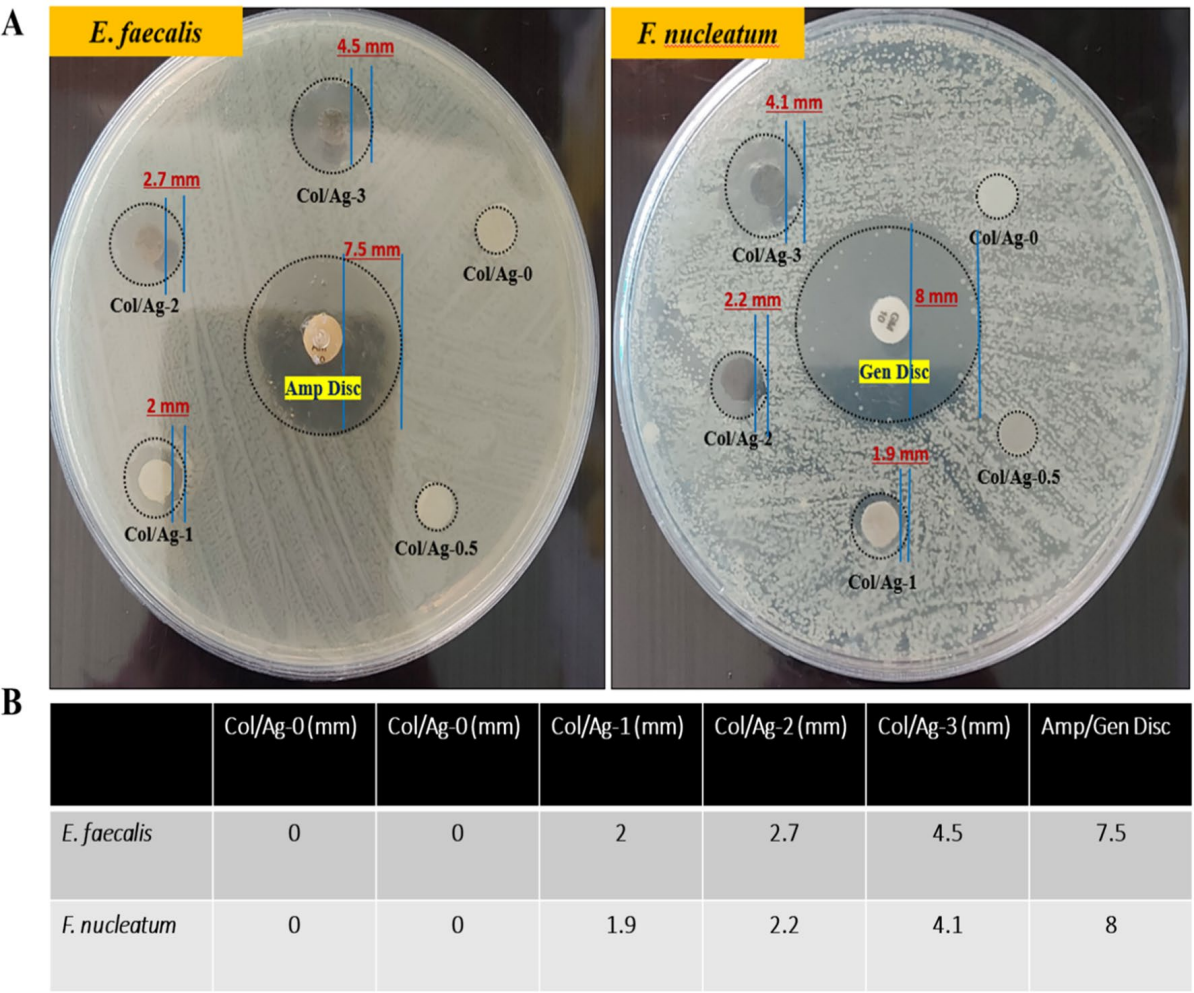
Antimicrobial properties of Col/Ag-0, Col/Ag-0.5, Col/Ag-1, Col/Ag-2, and Col/Ag-3 against *Fusobacterium nucleatum* and *Enterococcus faecalis*. (**A**) Photograph images of the zone of inhibition and (**B**) the diameters of the bacteriostatic zones of different samples.

The antibacterial activity of Col/Ag-0, Col/Ag-0.5, Col/Ag-1, Col/Ag-2, and Col/Ag-3 against *Fusobacterium nucleatum* and *Enterococcus faecalis* was evaluated using the inhibition zone method. The results are presented in Fig. 5A and B. It is evident from the results that Col/Ag-0 and Col/Ag-0.5 did not exhibit any inhibition zone against *Fusobacterium nucleatum* and *Enterococcus faecalis*. However, distinct bacterial-free zones surrounding the rounded Col/Ag-1, Col/Ag-2, and Col/Ag-3 samples were visible (indicated by black circles). Moreover, due to the higher content of AgNPs in Col/Ag-2 and Col/Ag-3, the size of the bacterial-free zones was larger compared to Col/Ag-1. In comparison to the control groups and Col/Ag-0.5, the presence of an inhibition zone in Col/Ag-1, Col/Ag-2, and Col/Ag-3 suggests the release of AgNPs or Ag^+^ ions, which provide antibacterial effects. This characteristic is particularly important for a GTR membrane in the treatment of periodontitis.

### Membrane-bacteria co-culture system

**Figure 6:**
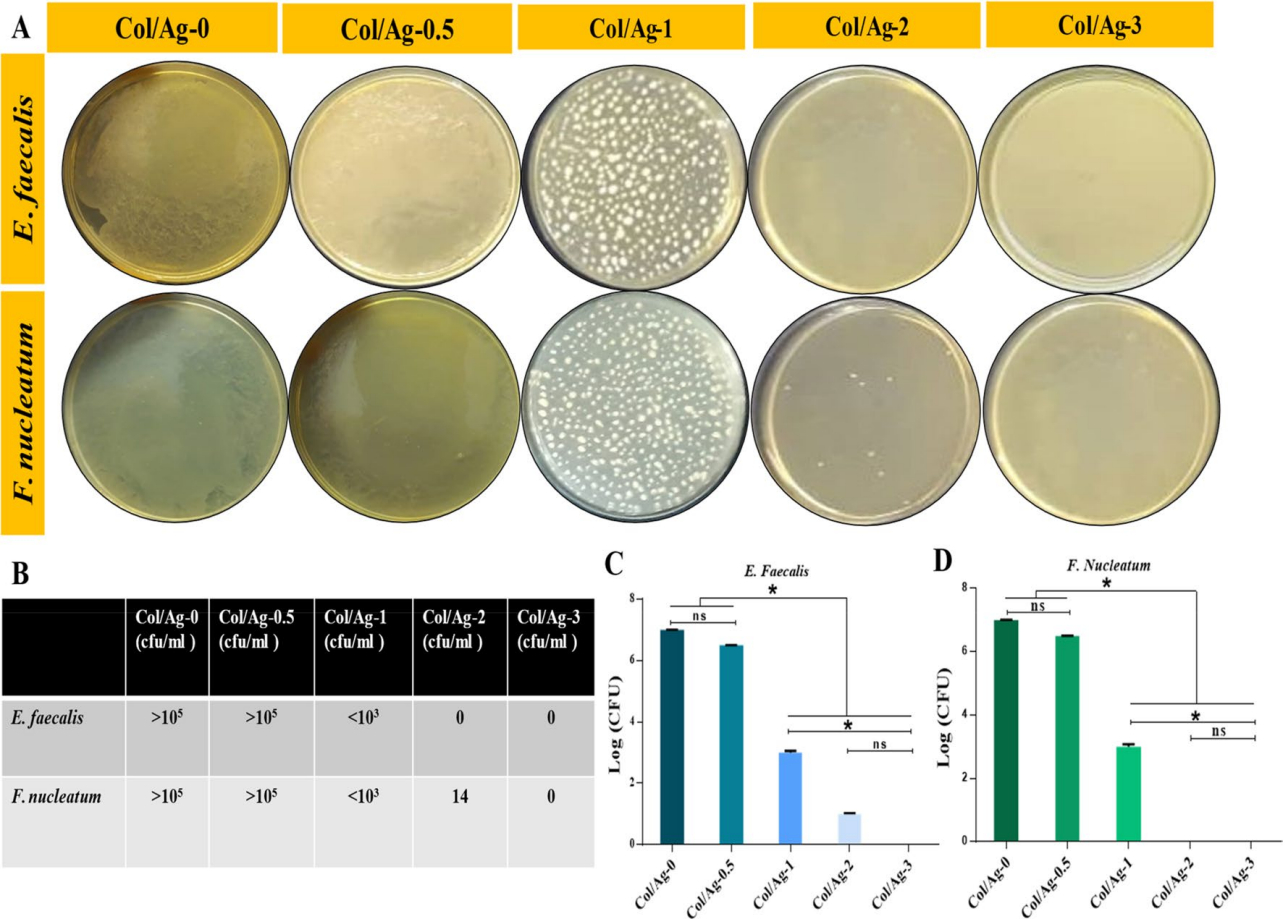
(**A**) Digital photos of agar plates with bacterial colony-forming units (CFU), (**B**–**D**) quantitative bacterial survival rate calculated from corresponding CFU. Statistical analysis was performed by one-way ANOVA followed by Tukey’s multiple comparison tests, ∗p < 0.05.

Membrane-bacteria co-culture was also used to test the antibacterial effectiveness of membranes containing AgNPs. After 24 h of incubation with membranes, bacterial suspensions were plated on the agar (Fig. 6). A strong relationship between bacterial colonies and the concentrations of loaded AgNPs in membranes was realized. The bacterial colonies on the Col/Ag-0.5 membrane were visible and it showed the same effect as the Col/Ag-0 membrane. There was no significant difference between the Col/Ag-0 group and Col/Ag-0.5. However, the number of colonies significantly decreased in Col/Ag-1, and hardly any colonies were observable in Col/Ag-2 and Col/Ag-3. Overall, there was no significant difference between the Col/Ag-2 and Col/Ag-3 groups, while there was a significant difference in the number of colonies grown in Col/Ag-1 compared to Col/Ag-2 and Col/Ag-3. Results demonstrate that the number of colonies decreases as the percentage of AgNPs in the membranes rises, and in concentrations of 2 and 3% AgNPs, hardly any colonies grow. According to these findings, collagen modified by AgNPs at a specific concentration has been found to exhibit antibacterial activity against both Gram-positive and Gram-negative bacteria.

### SEM of bacteria-membrane

**Figure 7:**
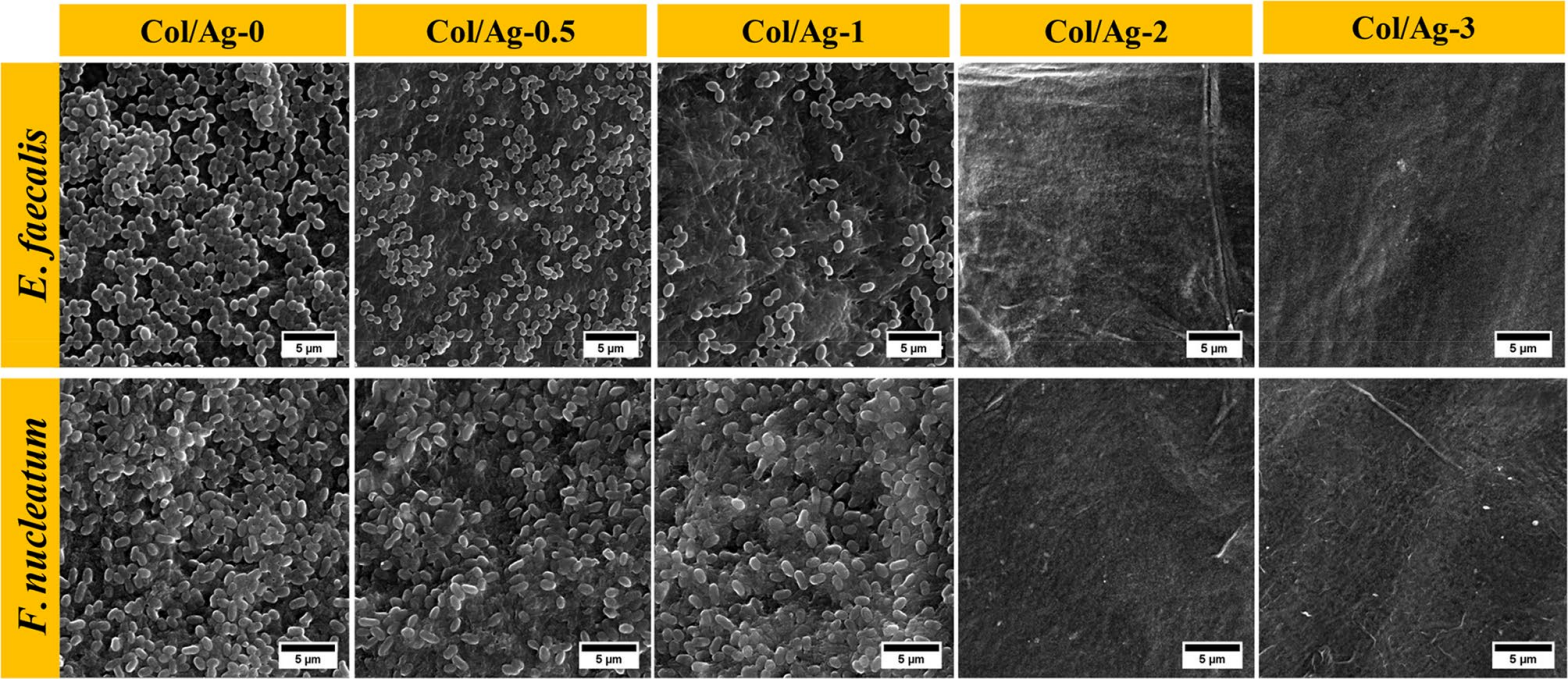
Antimicrobial properties of Col/Ag-0, Col/Ag-0.5, Col/Ag-1, Col/Ag-2, and Col/Ag-3 against *Fusobacterium nucleatum* and *Enterococcus faecalis*: SEM images of bacteria after incubation with membranes.

After being incubated for 24 h on AgNPs-incorporated collagen membranes, the morphology of *Enterococcus faecalis* and *Fusobacterium nucleatum* was observed by SEM as shown in Fig. 7. The bacteria on the Col/Ag-0 are in their natural spherical form (*Enterococcus faecalis*) and rod-shaped (*Fusobacterium nucleatum*) without any obvious damage to their structure. SEM images demonstrate that the bacteria grow well on the collagen membrane surface, have a plump morphology, and have not had their structural integrity compromised. Fewer microorganisms grew on the surface as the amount of AgNPs increased. Additionally, the integrity of the bacterial membrane started to break down, and many bacteria clumped together and displayed abnormal morphology. The growth of bacteria was completely inhibited in Col/Ag-2, and Col/Ag-3 compared to the Col/Ag-0.5 and Col/Ag-1, and no attached bacteria were seen on the observation membranes. Cellular debris of dead bacteria fell from the membrane surface after washing. The findings provide more evidence in favor of AgNPs antibacterial action, which allows for the destruction of cell membranes and eventual cell lysis^65,66^.

### Cytocompatibility

The in vitro cell compatibility of AgNPs-incorporated membranes was evaluated with HGF for 1, 3, and 7 days using live-dead assays, MTT assays, and cell attachment. These evaluations allow for a comprehensive understanding of the compatibility of Col/Ag membranes with HGF cells, ensuring their safety and effectiveness for potential biomedical applications.

### Cell viability and cell morphology

**Figure 8:**
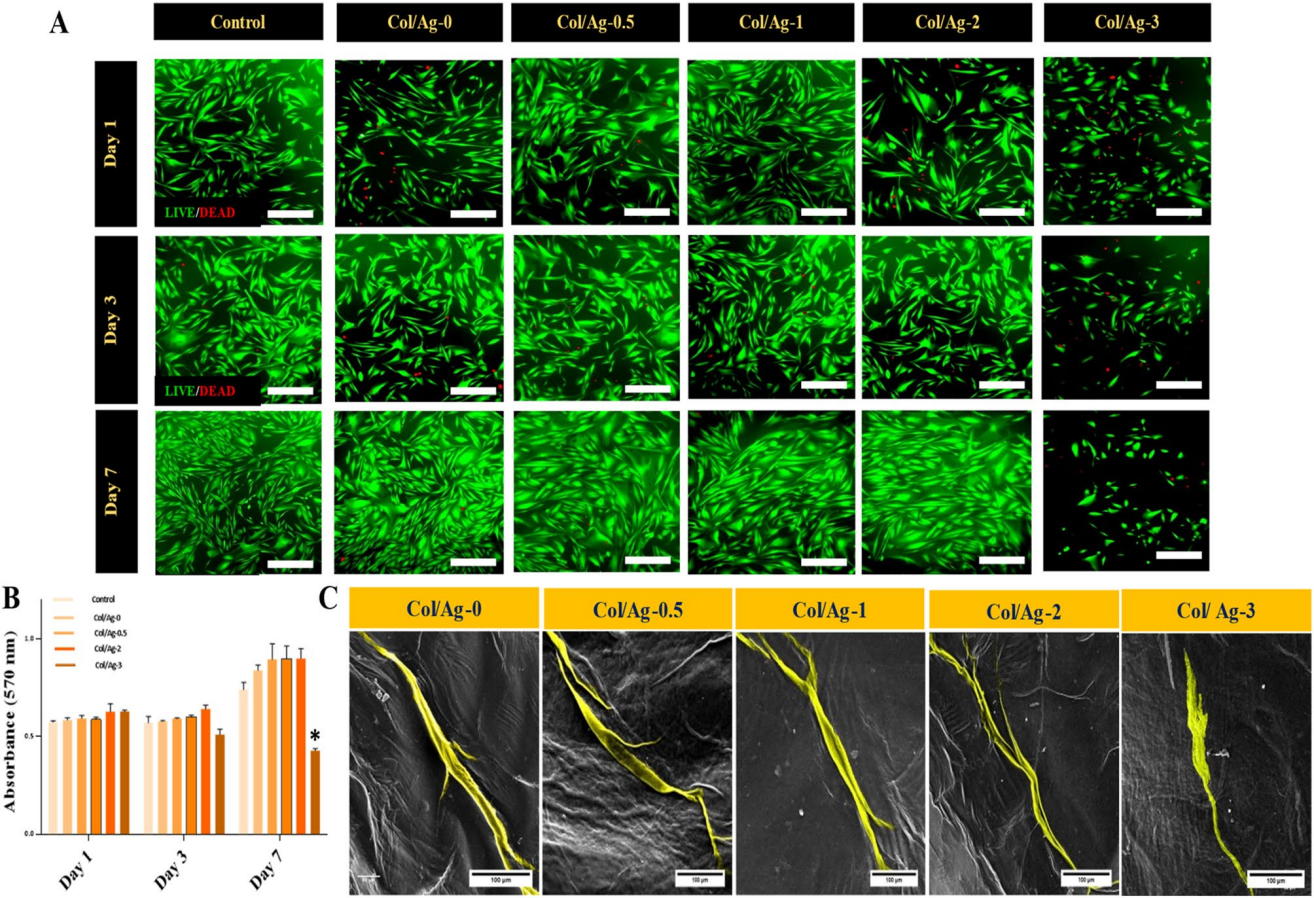
Evaluation of the cytocompatibility of membranes. (**A**) Fluorescent images of live/dead staining of human gingival fibroblasts (HGF) on plate (control), Col/Ag-0, Col/Ag-0.5, Col/Ag-1, Col/Ag-2, and Col/Ag-3 after culturing for 1, 3 and 7 days (Live cells were stained in green while dead cells were stained in red), Scale bar = 100 µm, (at a magnification of ×20) (**B**) Viability and proliferation of HGF cells on membranes measured using the MTT assay and (**C**) Cell morphology of HGF on membranes at day 3 observed under SEM, Scale bar = 100 µm. *Denotes a significant difference compared to the control group (p < 0.05).

The viability of cells on the membranes was evaluated using a Live/Dead assay. Figure 8A shows the live-dead images of the HGF cells, 1, 3, and 7 days after cultivation. The cell cultured on the plate well was taken as control. On day 1, fluorescence microscope micrographs revealed a higher number of live cells on the control, Col/Ag-0, Col/Ag-0.5, Col/Ag-1, and Col/Ag-2 membranes compared to the Col/Ag-3 membrane. After 3 days of cultivation, HGF cells proliferated in control, Col/Ag-0, Col/Ag-0.5, Col/Ag-1, and Col/Ag-2 membranes while fewer live cells were exhibited on the Col/Ag-3 membrane. After 7 days, the cultured cells on the control and Col/Ag-0, Col/Ag-0.5, Col/Ag-1, and Col/Ag-2 membranes have a significantly higher density than Col/Ag-3 membranes. In the membranes containing 0 to 2% silver, the morphology of the cells was similar to the control cells; they were shown as spindles and fully expanded, and with cell adhesion. However, in the membranes containing 3% silver, in addition to noticing that the number of cells decreased significantly, normal morphology of the cells was not seen.

### Cell proliferation

The impact of different percentages of AgNPs incorporated in membranes on HGF cell proliferation was evaluated using the MTT assay after 1, 3, and 7 days. As depicted in Fig. 8B, there was minimal variation in cell proliferation between the control group and the membranes on days 1 and 3 of incubation. The number of cells remained relatively constant during this period, with a slight increase observed on day 3, indicating cell proliferation. Notably, the proliferation of cells on the Col/Ag-3 membrane exhibited a statistically significant decrease compared to the other groups and the control.

### Cell attachment

SEM imaging was utilized to assess HGF cell attachment and morphology on the membranes. After 3 days of cultivation, cell-cultured membranes were washed with PBS, fixed in glutaraldehyde, dehydrated in ethanol, air-dried, and sputtered with gold for SEM observation. The morphologies of HGF cells on the Col/Ag-0, Col/Ag-0.5, Col/Ag-1, Col/Ag-2, and Col/Ag-3 membranes are depicted in Fig. 8C. HGF cells adhered to the surface of the Col/Ag-0, Col/Ag-0.5, Col/Ag-1, and Col/Ag-2 membranes, exhibiting a flattened morphology with highly mature elongated filopodia, closely resembling normal HGF morphology. Whereas, cells on the Col/Ag-3 membrane did not exhibit filopodia and showed weak spreading on the membrane. The microscopic images of the cells' morphology and the results of the live-dead test are consistent.

## Discussion

Pathogenic bacteria play a significant role in the development of oral diseases, particularly periodontitis, and can pose challenges to effective periodontal treatment. Collagen, the most abundant protein in the body, and is an excellent polymer for tissue regeneration^45^. However, collagen has several limitations in medicine for example, in addition to low mechanical properties, protein-based materials like collagen are prone to contamination by environmental microorganisms due to their highly hydrophilic and nutritive properties. To address this issue, it is common practice to incorporate appropriate antimicrobial agents into collagen membranes to confer antimicrobial properties^67^. AgNPs, renowned for their effective, broad-spectrum antimicrobial properties and non-drug resistance characteristics, are frequently utilized in the field of biomedical materials^68–70^. On the other hand, it was previously believed that AgNPs were only toxic to prokaryotic cells, but more recent research has shown that AgNPs can also be toxic to mammalian cells at specific concentrations and can negatively affect cell viability^68^. Hence, for clinical application, the implants should have good biocompatibility as well as antimicrobial capability, particularly in periodontitis.

Therefore, this research aims to investigate the antibacterial properties of Collagen/Ag membranes specifically for their application in periodontitis treatment and optimize the ideal percentage of AgNPs. By doing so, this study aims to contribute valuable insights to the field of periodontology, advancing our understanding of effective strategies for combating periodontal diseases. For this purpose, AgNPs were synthesized using ascorbic acid as a reducing agent and citric acid as a stabilizer. The UV–Vis absorption spectra and the FE-SEM imaging confirmed the formation of spherical AgNPs with an average diameter of 30 nm. The XRD patterns of AgNPs confirmed the crystalline nature of the synthesized nanoparticles, with the presence of peaks at 2θ = 38.2°, 44.2°, and 64.5°. An antibacterial regenerative membrane was synthesized using collagen and AgNPs in the form of hydrogel, then the hydrogel was compressed and dried to form a Col/Ag membrane. Collagen-based structures promote tissue regeneration due to their porous network and support angiogenesis, cell growth, and cell proliferation^71^. Membrane SEM images showed that AgNPs are uniformly embedded throughout the membrane, because, the aminophilic nature of AgNPs enables their physical binding to collagen molecules. The incorporation of nanoparticles into the collagen solution, followed by collagen hydrogel formation, ensured the homogeneous dispersion of AgNPs within the hydrogel. The inner cross-sectional regions of the Col/Ag membrane exhibited a fibrillated and heterogeneous interconnected structure with open porosity, featuring micro-scale pore sizes, which are suitable for cell functions. The FTIR spectra analysis of collagen and Col/Ag membrane revealed that the inclusion of AgNPs in the collagen membrane potentially disrupted the secondary structure of collagen. This disruption could be attributed to the interactions between the AgNPs and the collagen molecule. The favorable mechanical properties of the GTR membrane are necessary for periodontal tissue repair. Here, the compressed collagen/Ag membrane showed comparable mechanical properties to the two commercialized membranes.

As mentioned in several studies, collagen has poor mechanical properties in regenerative medicine applications^72,73^. Compressing collagen constructs is a promising method for improving their mechanical properties in tissue engineering applications. Studies have demonstrated that plastic compression can lead to collagen sheets with increased fibrillar density, resulting in improved cell interactions and mechanical properties^72,74^. According to the results of studies on the effect of compression on the mechanical properties of collagen, in this research, we used the collagen hydrogel compression process to make membranes with better mechanical properties.

**Figure 9:**
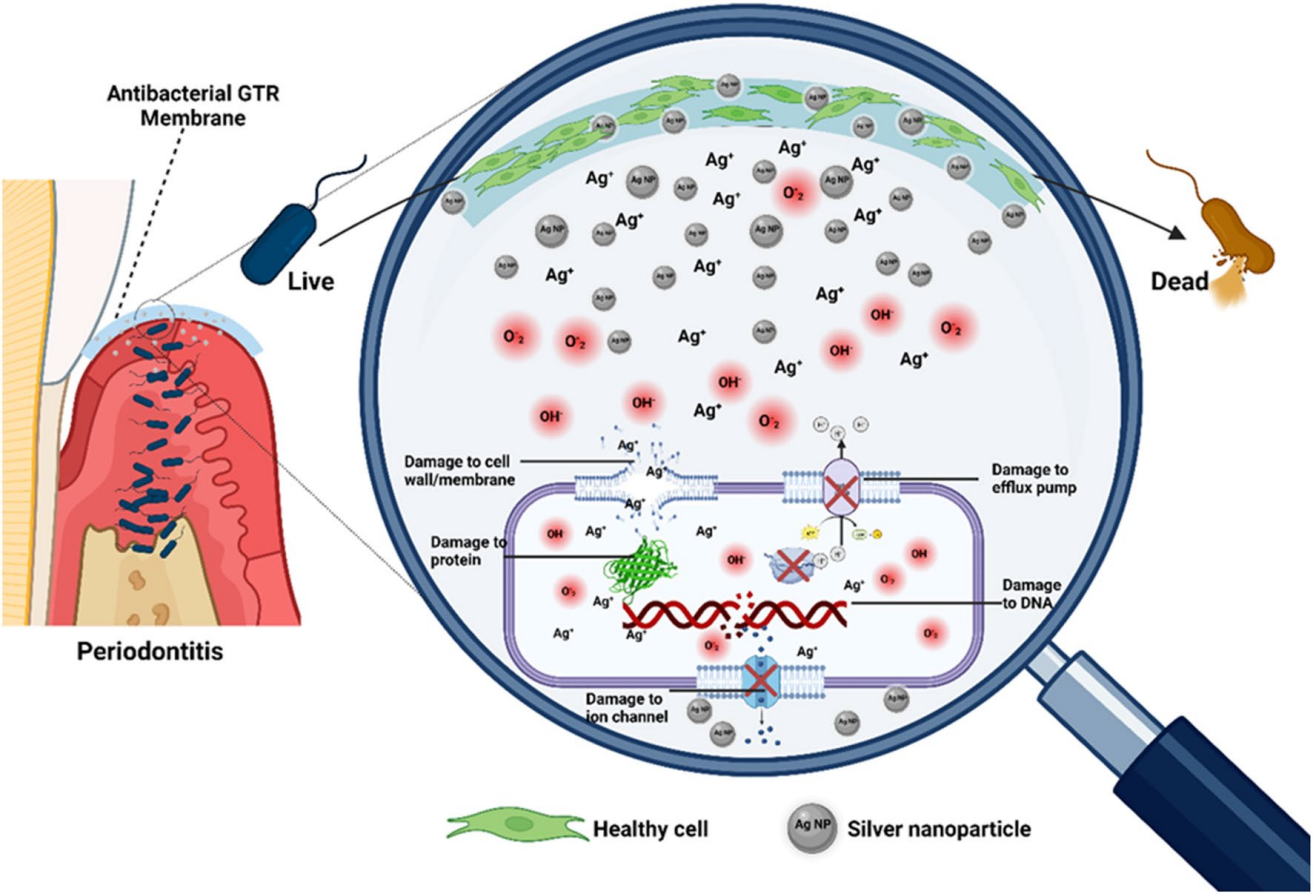
Schematic illustrating the antibacterial membranes with silver nanoparticles' mode of action in periodontitis.

Subsequently, the antibacterial assessment of the membrane demonstrated that the incorporation of 2% and 3% AgNPs effectively achieved their intended function. The antibacterial properties of AgNPs primarily rely on factors such as size, pH, ionic strength of the medium, and the concentration of AgNPs^75^. Released AgNPs ions penetrate the bacterial cell, leading to disturbances in its respiratory system and eventual cell death^76^. Additionally, AgNPs exhibit antibacterial effects by deactivating respiratory chain dehydrogenases and generating excessive reactive oxygen species (ROS), which hinder bacterial growth^77^. This mechanism is schematically illustrated in Fig. 9.

Subsequent to that, consistent results from cytotoxicity tests indicated that cells cultured on Col/Ag-0 to Col/Ag-2 membranes displayed excellent cell viability, proliferation, morphology, and adhesion. These findings align with previous studies that have confirmed the vital role of type I collagen, an extracellular matrix protein, in facilitating cell interaction by regulating cell attachment and proliferation^78^. The binding mechanisms between cells and collagen-based materials can be categorized as either integrin-mediated or non-integrin-mediated^79^. Integrins, which are transmembrane receptors, recognize and bind to specific amino acid sequences in the collagen molecule^79,80^. However, when comparing the Col/Ag-3 membrane to the other Col/Ag membranes (Col/Ag-0 to Col/Ag-2), noticeable differences in cell survival, proliferation, and attachment became evident as the culturing duration extended to 7 days. These unfavorable cellular responses observed on Col/Ag-3 can be attributed to the higher release rate of AgNPs from this membrane. It is important to note that the collagen membranes containing AgNPs were produced in a compressed form, and the compact structure may have gradually deteriorated, leading to an increased release rate of AgNPs or Ag^+^. Consequently, the cells were exposed to a higher concentration of AgNPs. It can be hypothesized that the incorporation of AgNPs into collagen membranes can regulate their release, enhancing antibacterial activity over time and providing a significant advantage in combating Bacteria^81^. It has been discovered that AgNPs induce cell death through lipid peroxidation, which disrupts the cell membrane^82^. The disruption of the cell membrane results in the detachment of specific components of the cell membrane, ultimately disturbing integrin-mediated adhesion and triggering apoptosis^83,84^.

## Conclusion

In this study, silver nanoparticles (AgNPs) were successfully synthesized using an environmentally friendly method and were incorporated into collagen membranes to create collagen/Ag membranes with varying AgNPs content. The antibacterial and cytotoxic properties of these membranes were extensively investigated to evaluate their potential as effective agents in periodontitis treatment strategies. Our results demonstrated that among the five different samples tested, the collagen/Ag-2 and collagen/Ag-3 membranes exhibited superior antibacterial activity against both Gram-positive and Gram-negative bacteria, surpassing collagen/Ag-0.5, collagen/Ag-1, and the control (membrane without AgNPs). This highlights the significant enhancement of the membranes' antibacterial properties upon the incorporation of silver nanoparticles, making them promising candidates for combating periodontitis, a common oral inflammatory condition caused by bacterial accumulation in the gum tissues. Interestingly, we observed a distinction in cytocompatibility between collagen/Ag-2 and collagen/Ag-3. Collagen/Ag-2 demonstrated better compatibility with mammalian cells, suggesting reduced potential for harm or toxicity compared to collagen/Ag-3. This finding is crucial as it indicates that while both formulations possess potent antibacterial properties, collagen/Ag-2 might be more suitable for periodontitis treatment due to its reduced impact on healthy mammalian cells during therapy. Overall, our study highlights the remarkable potential of collagen/Ag membranes, specifically collagen/Ag-2, as a viable treatment option for periodontitis. The incorporation of 2% w/w AgNPs into the collagen membrane, a biocompatible and natural polymer, offers an encouraging strategy for effectively combating bacteria without inducing cytotoxicity in mammalian cells. These findings contribute valuable insights to the field of periodontal therapy and provide a promising foundation for developing advanced biomaterials aimed at improving periodontal treatment outcomes.

## Data Availability

The data that support the findings of this study are available from the corresponding author, Dr. Esmaeil Mirzaei, upon reasonable request.
